# Upregulation of triglyceride synthesis in skeletal muscle overexpressing DGAT1

**DOI:** 10.1186/1476-511X-12-63

**Published:** 2013-05-04

**Authors:** Feifei Yang, Zhuying Wei, Xiangbin Ding, Xinfeng Liu, Xiuguo Ge, Guimin Song, Guangpeng Li, Hong Guo

**Affiliations:** 1 Department of Animal Science, TianJin Agricultural University, TianJin, 300384, P. R. China; 2 Key Laboratory of National Education Ministry for Mammalian Reproductive Biology and Biotechnology, Inner Mongolia University, Hohhot, 010021, P. R. China

**Keywords:** DGAT1, Transgenic mice, Overexpression, Intramuscular fat content

## Abstract

The gene encoding diacylglycerol acyltransferase (*DGAT1*) is a functional and positional candidate gene for milk and intramuscular fat content. A bovine DGAT1 overexpression vector was constructed containing mouse MCK promoter and bovine DGAT1 cDNA. MCK-DGAT1 transgene in FVB mice was researched in present study. The transgene DGAT1 had a high level of expression in contrast to the endogenous DGAT1 in posterior tibial muscle of the transgenic mice, but a low expression level in the cardiac muscle. Compared with wild-type mice, triglyceride and DGAT1 content were approximately fourfold and 50% increased in posterior tibial muscle of the transgenic mice, respectively, while a little increase in cardiac muscle.

## Background

Triacylglycerols (TG) are quantitatively the most important storage form of energy for eukaryotic cells. Diacylglycerol acyltransferase 1 (*DGAT1*) encodes acyl CoA:diacylglycerol acyltransferase (DGAT) which catalyzes the terminal and only committed step in triacylglycerol synthesis [1]. DGAT1 is present in all cell types but is most highly expressed in tissues and organs where triglyceride synthesis is most active, including adipose tissue, liver, skeletal muscle and small intestine. Bovine *DGAT1* spaning 14117 base pairs (bp) was assigned to a region on bovine chromosome 14 close to the centromere and contains 17 exons and 16 introns [2]. Its coding sequence (CDS) spans 1470 bp and encodes 489 amino acids. DGAT deficiency alters triglyceride metabolism in mammary gland of mice [3]. Thaller *et al*. [4] treated *DGAT1* as a positional and functional candidate gene for intramuscular fat deposition in cattle. Myocellular overexpression of DGAT1 resulted in increased intramyocellular TG levels but decreased myocellular DAG and ceramide levels [5].

Muscle creatine kinase (MCK) is critical to the energy metabolism of skeletal muscle as the key enzyme in the phosphorylcreatine shuttle between mitochondria and the myofibrils [6]. The MCK gene is transcriptionally activated during striated muscle differentiation and is expressed at high levels in skeletal and cardiac muscles. Transcriptional regulation of the gene in cultured skeletal and cardiac muscle cells has been shown, as well as in transgenic mice [7]. Jaynes *et al*. [8] demonstrated a 3.3 kilobases (kb) 5′-flanking region of the MCK gene which was sufficient to confer transcriptional regulation to a heterologous structural gene (chloramphenicol acetyl transferase). The major effector of high-level expression was found to have the properties of a transcriptional enhancer. This element, located between 1050 and 1256 bp upstream of the transcription start site, activated either the MCK promoter or heterologous promoters in differentiated muscle cells [9]. Besides the 206 bp MCK enhancer, the 1 kb region of DNA between the enhancer and the basal promoter is essential for the transgenic expression in skeletal muscle ,unlike its behavior in cell culture [10]. Long terminal repeat (LTR) inserted with MCK enhancers was utilized by Wang *et al*. [11] to increase human factor IX (FIX) expression in skeletal muscle cells in vitro and in vivo.

In the present study, we wanted to investigate whether bovine *DGAT1* could express in skeletal muscle of mice under the control of mouse MCK promoter, and determine the effect of bovine DGAT1 overexpression on triglyceride synthesis and storage in MCK-DGAT1 transgenic mice.

## Results

### Overexpression of DGAT1 in skeletal muscle of MCK-DGAT1 transgenic mice

**Figure 1:**
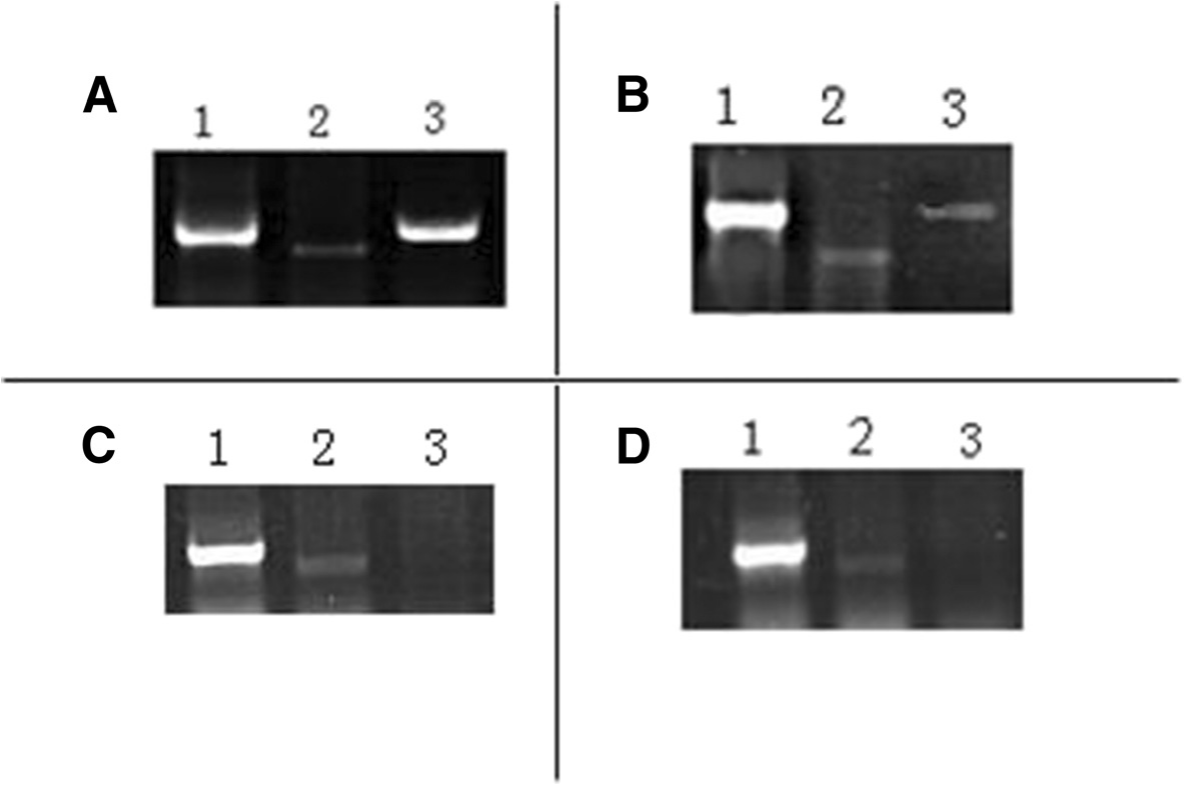
**Expression level of endogenous DGAT1 and transgene in five transgenic mice and five WT mice.** (**A**) Expression in posterior tibial muscle of one transgenic mice. (**B**) Expression in cardiac muscle of one transgenic mice. (**C**) Expression in posterior tibial muscle of one WT mice. (**D**) Expression in cardiac muscle of one WT mice. (1) The expression of Gapdh. (2) The expression of endogenous DGAT1. (3) The expression of transgene DGAT1.

We used the 1.38 kb mouse MCK promoter, which is widely used for skeletal and cardiac muscle transgene expression, to direct the expression of a full-length bDgat1 cDNA. The RT gene expression analysis was performed on five transgenic mice and five WT mice, which showed that expression of the transgene was primarily restricted to skeletal muscle, and had a high level contrast to the endogenous DGAT1 in posterior tibial muscle of the transgenic mice (Figure 1A), but a very low expression level in the cardiac muscle (Figure 1B). Gene expression in one WT mice were showed in Figure 1C and Figure 1D.

### Increased TG and DGAT1 content in skeletal muscle of MCK-DGAT1 transgenic mice

**Figure 2:**
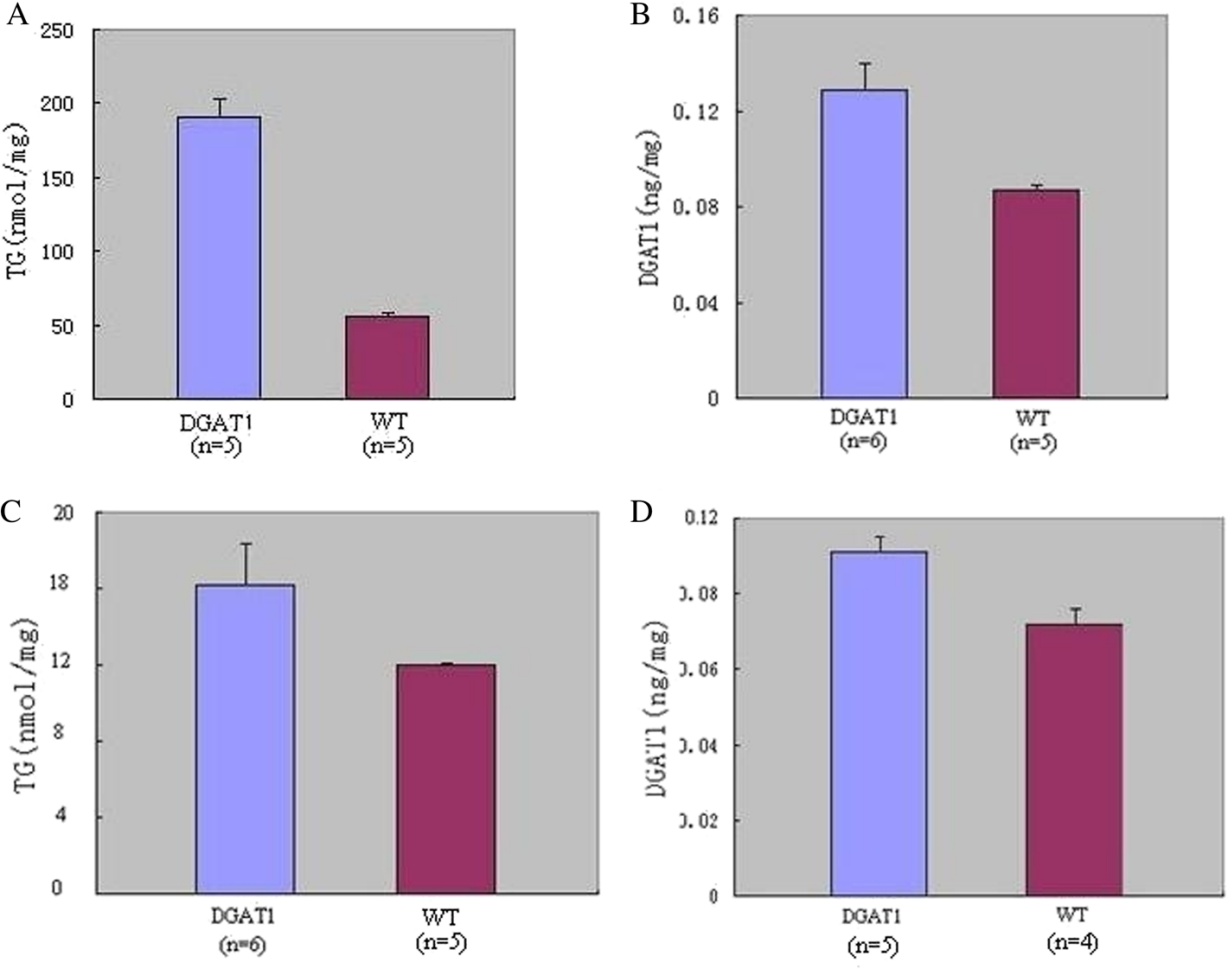
**TG and DGAT1 levels in posterior tibial and cardiac muscle of MCK-DGAT1 mice and WT mice.** (**A**) TG content in posterior tibial muscle of MCK-DGAT1 mice (190.05 ± 13.17 nmol/mg) was significantly higher than that of WT mice (56.36 ± 2.29 nmol/mg, P<0.01). (**B**) DGAT1 content in posterior tibial muscle of MCK-DGAT1 mice (0.129 ± 0.011 ng/mg) was significantly higher than that of WT mice (0.087 ± 0.002 ng/mg, P<0.01). (**C**) TG content in cardiac muscle of MCK-DGAT1 mice (16.21 ± 2.14 nmol/mg) was significantly higher than that of WT mice (12.00 ± 0.09 nmol/mg, P<0.01). (**D**) DGAT1 content in cardiac muscle of MCK-DGAT1 mice (0.091 ± 0.004 ng/mg) was significantly higher than that of WT mice (0.072 ± 0.004 ng/mg, P<0.01).

10 – 12 weeks mice were used in this study. No significant difference in body weight was detected between the transgenic (19.57 ± 1.04 g) and WT (20.03 ± 1.31 g) mice. TG levels in posterior tibial muscle were approximately fourfold increased in MCK-DGAT1 mice compared with WT mice (Figure 2A). A approximately 50% increase in total DGAT content was observed in posterior tibial muscle of the transgenic mice, compared with WT mice (Figure 2B). TG (Figure 2C) and DGAT1 (Figure 2D) content in cardiac muscle of MCK-DGAT1 mice were only a little higher than that of WT mice.

## Discussion

MCK-DGAT1 transgenic mice were healthy and fertile. The present study shows that bovine *DGAT1* could express in skeletal muscle of mice under the control of mouse MCK promoter, and overexpression of DGAT1 resulted in increased TG levels in skeletal muscle of mice, while the influence of transgene on cardiac muscle was low. Liu *et al*. [5] demonstrated similar results in skeletal muscles including gastrocnemius, soleus, and posterior tibial muscles, and heart. Their results also showed the effect of DGAT1 on muscle insulin sensitivity and that DGAT1 transgenic mice were resistant to HFD (high fat diet) – induced obesity. Thus, the overexpression of DGAT1 is theoretically a possible means to protect the healthy of transgenic animals while increasing intramuscular fat.

Intramuscular fat (percentage of lipid content of muscle), also subjectively assessed as marbling, represents an important beef quality trait [4]. The MCK-DGAT1 transgene is expected to applying in bovine to improve the quality of beef. In the subsequent study, we will investigate whether the mouse MCK promoter works in bovine.

Liu *et al*. [12] first demonstrated in vivo that although skeletal muscle of MCK-DGAT1 mice was more resistant to developing HFD-induced lipotoxicity, their liver was as susceptible for lipotoxic insulin resistance as that of wild-type mice. In our study, fatty liver was not observed in transgenic mice. Further researches will performed in subsequent study.

## Methods

### Generation of MCK-DGAT1 transgenic mice

**Figure 3:**
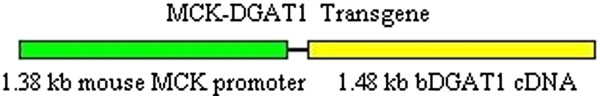
**The MCK-DGAT1 transgene.** The transgene contains, from the 5′-end to the 3′-end, an 1.38 kb mouse MCK promoter, and an 1.48 kb full-length bovine DGAT1 (bDGAT1) cDNA.

The transgene contains, from the 5′-end to the 3′-end, an 1.38 kb mouse MCK promoter, and an 1.48 kb full-length bovine DGAT1 (bDGAT1) cDNA (Figure 3). Transgenic mice were produced by pronuclear injection of FVB fertilized eggs with the transgene. Genotyping was carried out by PCR using genomic DNA extracted from mouse tails. The PCR was performed with primers F (5′- GATCCCCTATGGTGCACTCT -3′) and R (5′- TACGTCTCCGTCCTTGTCTG -3′) that amplified an 1.8 kb fragment in MCK-DGAT1 mice.

### Test conditions

Wild-type (WT) mice were purchased. Mice were housed in a barrier facility with 12-hour light/12-hour dark cycles and fed normal chow.

### Reverse transcriptase PCR

Total RNA was extracted from cardiac and posterior tibial muscle using TRIzol Reagent purchased from Invitrogen. The muscle were first homogenized with TRIzol Reagent, then RNA was separated in chloroform, precipitated by isopropanol, and washed by 75% ethanol, lastly the RNA was dissolved in RNase-free water. Total RNA was reverse-transcribed into cDNA which was used in the subsequent PCR amplification. The mRNA levels were quantified using the following primers: mouse endogenous DGAT1, forward, 5′- GCCCAAGGTAGAAGAGGACGAG-3′, reverse, 5′- AGTATGATGCCAGAGCAAACACG-3′, PCR temperature program, 95°C 3 min, then 95°C 30 s, 59°C 30 s, 72°C 30 s for 30 cycles, 72°C 5 min; transgene DGAT1, forward, 5′- AGAGGAGGTGCGGGATGTGG-3′, reverse, 5′- TCGCGGTAGGTCAGGTTGTCG-3′, PCR temperature program, 95°C 3 min, then 95°C 30 s, 61°C 30 s, 72°C 30 s for 30 cycles, 72°C 5 min. mRNA levels were then expressed as ratios relative to Gapdh, which express stably in this test, forward, 5′- GATGCCCCCATGTTTGTGAT-3′, reverse, 5′- GGGTGGTCCAGGGTTTCTTA-3′, PCR temperature program, 95°C 3 min, then 95°C 30 s, 54°C 30 s, 72°C 30 s for 30 cycles, 72°C 5 min. The RT gene expression analysis was performed on five transgenic mice and five WT mice.

### TG content

Tissues were homogenized by pestles in isoamyl alcohol. Triglyceride mass in lipid extracts was enzymatically determined with a colorimetric kit.

### DGAT1 content

Tissues were homogenized by pestles in PBS. Total DGAT1 content was measured with a DGAT1 ELISA kit. Double antibody sandwich ELISA is used in this kit to measure the content of DGAT1 in tissue homogenate quantitatively.

### Statistical analysis

Data were expressed as means ± SD. Statistical differences were analyzed by ANOVA to determine overall treatment effects including TG and DGAT1 content (SPSS 11.0). A two-tailed P value of 0.01 was used to indicate statistical significance.

### Ethical approval

The experiment was conducted according to protocols approved by the Institutional Animal Care and Use Committee at Tianjin Agriculture University.

## Competing interests

The authors declare that they have no competing interests.

## Authors’ contributions

FY carried out the whole experiment of the studies and drafted the manuscript. ZW, GL, HG and GS participated in the design of the study. XD, XL and XG participated in the experiment and helped to the data analysis. All authors read and approved the final manuscript.

## Authors’ information

Zhuying Wei Joint first author.
